# Non-symbolic Ratio Reasoning in Kindergarteners: Underlying Unidimensional Heuristics and Relations With Math Abilities

**DOI:** 10.3389/fpsyg.2022.800977

**Published:** 2022-02-11

**Authors:** David Muñez, Rebecca Bull, Pierina Cheung, Josetxu Orrantia

**Affiliations:** ^1^Centre for Research in Child Development, National Institute of Education, Nanyang Technological University, Singapore, Singapore; ^2^Macquarie School of Education, Macquarie University, Sydney, NSW, Australia; ^3^Faculty of Education, University of Salamanca, Salamanca, Spain

**Keywords:** ratio reasoning, preschool children, mathematics, non-symbolic, ANS

## Abstract

Although it is thought that young children focus on the magnitude of the target dimension across ratio sets during binary comparison of ratios, it is unknown whether this is the default approach to ratio reasoning, or if such approach varies across representation formats (discrete entities and continuous amounts) that naturally afford different opportunities to process the dimensions in each ratio set. In the current study, 132 kindergarteners (Mage = 68 months, *SD* = 3.5, range = 62–75 months) performed binary comparisons of ratios with discrete and continuous representations. Results from a linear mixed model revealed that children followed an additive strategy to ratio reasoning—i.e., they focused on the magnitude of the target dimension across ratio sets as well as on the absolute magnitude of the ratio set. This approach did not vary substantially across representation formats. Results also showed an association between ratio reasoning and children’s math problem-solving abilities; children with better math abilities performed better on ratio reasoning tasks and processed additional dimensions across ratio sets. Findings are discussed in terms of the processes that underlie ratio reasoning and add to the extant debate on whether true ratio reasoning is observed in young children.

## Introduction

Although rational numbers are usually introduced at late stages in elementary education, children engage in ratio reasoning well before the onset of formal school. Deciding whether extra cheese and pepperoni are needed to get the perfect pizza or choosing among different bowls of rice with veggies are frequent instances of ratio reasoning in children. Nonetheless, it is not yet clear that such instances reflect a true understanding of proportions and ratios as young children tend to confound ratio and absolute magnitude. For instance, when presented with two different jars containing blue and yellow beads (each jar reflecting different ratios of blue to yellow) and tasked to select the jar for which the probability of getting a blue bead is higher, 6-year-old children usually select the jar containing more blue beads, independently of the ratio blue to yellow in each jar (Falk et al., 2012). It is thought that such an error is a “conceptual misunderstanding” that reflects children’s inability to establish part–whole relations between the parts or dimensions of a ratio set (i.e., each jar) and that they rely on unidimensional heuristics (e.g., comparing the absolute magnitude of the target dimension across ratio sets). In the current study, we focus on how kindergarten children (6-year-olds) reason about ratios with continuous and discrete representations (i.e., reasoning about second-order relations; Spinillo and Bryant, 1991). Specifically, we look at whether children rely on specific unidimensional heuristics to reason about ratios and whether this approach is consistent across different representation formats (continuous vs. discrete) that naturally afford different opportunities to establish part–part relations and part–whole relations. Furthermore, we investigate the association between kindergarteners’ ratio reasoning skills and their math abilities because such reasoning skills are thought to function as a scaffold for symbolic ratio reasoning once children enter formal education (Szkudlarek and Brannon, 2021). Indeed, probability (probabilistic reasoning) and data analysis are core mathematical aspects to be developed from Pre-K2 to Grade 12 according to the National Council of Teachers of Mathematics (2000).

### Non-symbolic Ratio Reasoning in Children: An Approximate and Intuitive Ability

There is evidence that ratio sensitivity emerges early in development. For instance, McCrink and Wynn (2007, see also Téglás et al., 2007, 2011, Xu and Garcia, 2008) found that even pre-verbal infants can detect changes in ratio. Using a dishabituation paradigm with different ratios of arrays of blue and yellow dots, they found that 6-month-old infants were able to discriminate ratio differences between 2:1 and 4:1. In a series of experiments, Denison and Xu (2014, see also, Denison et al., 2013) found that 10–12-month-old infants were able to pick a cup that contained a lollypop drawn from a jar with a more favorable ratio of their preferred color.

Studies tackling the development of ratio reasoning^1^ in preschool and school-age children—many of them inspired by the seminal work of Piaget and Inhelder (1951) on probabilistic reasoning— have contributed to that evidence of ratio reasoning using a wide variety of paradigms and experimental contexts. Children as young as 3–4-year-old can match ratio displays that do not share representational or referential properties. For instance, they understand shape analogies such as matching 1/3 of a triangle and 1/3 of a square (e.g., Goswami, 1989, 1995; see also Spinillo and Bryant, 1991; Sophian, 2000). At about that same age, children can also transcode or map proportions across different formats. For instance, Möhring et al. (2016; see also Möhring et al., 2018; Gouet et al., 2020), presented 4–5-year-old children with different relative amounts of water and cherry juice (represented as blue and red rectangles) and asked them to rate the taste of these mixtures; children were able to approximate the ratio represented by each display (the taste of the mixture) on a non-labeled “number” line. Older children can solve more complex ratio reasoning tasks. For instance, Boyer and Levine (2012, see also Spinillo and Bryant, 1991; Boyer et al., 2008; Hurst and Cordes, 2018) found that school-age children (9--10-year-olds) can scale up (and down) proportions^2^ (e.g., matching equivalent proportional relations such as two stacked bar graphs of different lengths representing the same ratio), which is relevant in scaffolding more formal (symbolic) mathematical understanding. Note that whereas there is evidence that younger children can perform above chance on similar proportional reasoning tasks, there are multiple factors that affect performance (e.g., scaling, format of representation, and reasoning complexity). For instance, He et al. (2018, Experiment 1) found that 5–6-year-olds were not able to match equivalent proportions when tasked to assess whether two different stacked bars (representing the same proportion of juice and water) were equivalent if bars differed in height; nonetheless, they performed above chance when bars differed in width. Similarly, Yang and He (2021) investigated ratio reasoning abilities in 4–6-year-olds and found that younger children failed to reason about ratios when three-dimensional pictures were shown.

### True Ratio Reasoning vs. Unidimensional Heuristics

Although the studies mentioned above show that preschool and kindergarten children perform above chance in ratio reasoning tasks, it is not clear whether young children engage in true ratio reasoning (i.e., establishing part–whole relations between dimensions of each ratio set), or they focus on unidimensional aspects across ratio sets instead. That is, focusing on the target dimension (i.e., preferred color, flavor, etc.) across ratio sets using a “more-good strategy,” focusing on the non-target dimension across ratio sets using a “less-good strategy,” or focusing on differences in the absolute/total magnitude of ratio sets using a “more items strategy.” This is because the target ratio and the absolute magnitude of the target dimension across ratio sets are not usually disentangled in experimental designs and data analyses. For instance, early studies with infants and preschool children have predominantly presented children with stimuli in which the side of the target ratio corresponded to the side showing the larger target dimension (for a review, see Falk et al., 2012). Hence, children could respond to the target ratio by simply focusing on the target dimension across ratio sets (establishing part–part relations) rather than establishing part–whole relations between the dimensions in each ratio set and assessing the ratio of each ratio set (i.e., processing the ratio of ratios rather than the ratio of the target dimension across ratio sets). Indeed, this seems the default approach to ratio reasoning in preschool and kindergarten children (e.g., Siegler, 1981; Falk and Lann, 2008; Falk et al., 2012), which may be deemed as a primitive approach in the sense that it is neglected that each ratio set is determined by the relation between target and non-target dimensions. Studies with older children have revealed that they have a better understanding of that association between dimensions in a ratio set. For instance, Falk et al. (2012, see also Szkudlarek and Brannon, 2021) found that by the age of 7 children focus on both the target and non-target dimensions across ratio sets when tasked to choose among two different jars containing blue and yellow beads.

### Continuous vs. Discrete Representations

Whereas preschool children and kindergarteners (4–6-year-olds) typically struggle with discrete representations such as arrays of dots, beads, or marbles (in experiments that avoid confounding target ratio and magnitude of the target dimension, e.g., Falk et al., 2012), they perform above chance with continuous representations such as comparing the ratio of a pair of stacked bar graphs (e.g., Jeong et al., 2007). It has been suggested that younger children struggle with discrete representations because of difficulties in coding each of the relevant dimensions (Sophian and Wood, 1997; see also Singer and Resnick, 1992). For instance, assessing the ratio of blue to red beads in a jar is more challenging than estimating the ratio of oil to water in a glass because the absolute magnitude of each dimension in continuous representations is defined by physical boundaries. It is thought that the visual system extracts real or virtual axes of symmetry and that, together with object’s boundaries, serve as points of reference for proportional processing (Spence, 1990). Thus, the boundary of both dimensions in continuous representations, e.g., a stacked bar graph—would afford estimation of the proportion of the target dimension (part–whole structures); in contrast, discrete representation such as a random array of blue and red dots would pose perceptual constraints to determine adequate referential points and identify the parts and the whole (Mix et al., 2002).

Relatedly, there is evidence that the cognitive systems involved in processing visual properties that represent ratios develop earlier for continuous magnitudes such as length and space than for discrete quantities (note that it is out of the scope of this paper whether these systems represent the same entity or not; for a discussion, see Bueti and Walsh, 2009). For instance, Kucian et al. (2018) presented school-age children (P3–P6) with two different tasks in which children had to compare arrays of dots and angle sizes (represented as a pair of Pac-Man with different mouth angles) and found that angle processing was easier across the entire age-range. Kucian et al. (2018) also found that performance on both tasks decreased as a function of difficulty with the ratio being processed, which aligns with findings from ratio reasoning tasks (e.g., Szkudlarek and Brannon, 2021). These studies underscore the role of the cognitive systems involved in processing and representing ratios and how these systems’ limitations (or accuracy) may affect ratio reasoning. For instance, the *approximate number system* (ANS), which is thought to afford binary decisions involving magnitude comparison tasks when discrete representations are used (for comprehensive reviews, see Odic and Starr, 2018), is a predictor of ratio reasoning abilities (Szkudlarek and Brannon, 2021).

### Non-symbolic Ratio Reasoning and Children’s Math Abilities

The fact that non-symbolic ratio representations are indeed ratios that can be defined with symbol numbers naturally sets the grounds for a plausible link between non-symbolic ratio reasoning and symbolic ratio reasoning—fraction understanding. Indeed, grasping the concept of fractions involves proportional reasoning strategies (Fuchs et al., 2013). It has been underscored that having an understanding of both probability reasoning and data analysis is a mathematical goal in elementary education (National Council of Teachers of Mathematics, 2000). Despite this theoretical link and educational relevance, few studies have provided empirical evidence of an association between ratio reasoning and math abilities. For instance, Matthews et al. (2016) found a moderate (positive) correlation between non-symbolic ratio processing in the context of a ratio comparison task and performance on a fraction magnitude comparison task in college students. Jordan et al. (2017) found that non-symbolic ratio reasoning in fifth grade was a predictor of fraction understanding 1 year later (see also Möhring et al., 2016). Evidence of that association with younger children is very limited because fractions are only introduced in late elementary school. Thus, studies with younger children have reported links with other math aspects. For instance, Szkudlarek and Brannon (2021) found that non-symbolic ratio reasoning with discrete representations was related to performance on the Key-Math-3 Numeration subtest in 6–7-year-olds (although this association vanished after accounting for individual differences in ANS acuity) and the Key-Math-3 Data Analysis and Probability subtests—which is a test that includes proportional reasoning aspects.

That link between ratio reasoning and fraction understanding is also supported by studies that have specifically investigated whether ratio reasoning can be harnessed and whether improvements in ratio reasoning translate to improvements in math and fraction understanding. For instance, Szkudlarek and Brannon (2021) found that non-symbolic ratio reasoning may function as a scaffold for symbolic ratio reasoning in children who lack an understanding of fractions (see also Gouet et al., 2020; Abreu-Mendoza et al., 2021). Other training studies involving mapping non-symbolic continuous representations of proportions with fractions have resulted in improvements in fraction knowledge in older children (Fazio et al., 2016; Soni and Okamoto, 2020).

### The Current Study

Although it has been suggested that young children rely on unidimensional heuristics to reason about ratios —specifically, they tend to focus on the magnitude of the target dimension across ratio sets (Jeong et al., 2007; Falk et al., 2012)—few studies have investigated whether this is the default approach to ratio reasoning when children are presented with discrete entities and continuous amounts. The bulk of evidence with young children is based on experimental designs in which the possibility that unidimensional heuristics explain how children behave has not been accounted for. Furthermore, few studies have specifically contrasted young children’s ratio reasoning performance on both discrete (with uncountable representations) and continuous tasks. Indeed, the bulk of evidence on children’s ratio reasoning skills with discrete representations comes from studies that have investigated the so-called *numerical interference* in proportional reasoning or *whole number bias*—i.e., children tend to count instead of focusing on the relations between the dimensions of a ratio set and across ratio sets when proportions are presented discretely with distinct units that can be explicitly counted, like a rectangle divided into quarters (Ni and Zhou, 2005; Jeong et al., 2007; Boyer et al., 2008; Hurst and Cordes, 2018). Thus, the first goal of the current study is twofold: first, to determine whether kindergarteners’ ratio reasoning skills are based on specific unidimensional heuristics, and second, to examine whether children’s approach to ratio reasoning varies as a function of representation format. The literature review suggests that children’s approach to ratio reasoning may vary across representation formats because continuous and discrete representations naturally afford different opportunities to estimate the absolute magnitude of each dimension within each ratio set and across ratio sets. For instance, it is feasible that continuous representations allow additive approaches (e.g., focusing on the magnitude of both target and non-target dimension) since each dimension is perceptually more salient than in discrete representations.

**FIGURE 1:**
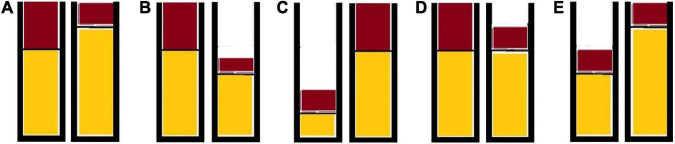
Examples of trial types according to unidimensional heuristics that can be used.

To test this hypothesis, children in the current study were presented with pairs of continuous and discrete ratio sets—reflecting two different mixtures—and tasked to select the most favorable ratio set. In the continuous condition, two stacked bar graphs representing two glasses containing a mix of milk and chocolate were shown. In the discrete condition, two glasses containing dots of different colors (representing chocolate and banana chips) were presented. We manipulated the magnitude of each dimension in each ratio set (and across ratio sets) to minimize the association between the target ratio and each unidimensional heuristic (i.e., the target dimension, the non-target dimension, and the absolute or total magnitude of each ratio set). Figure 1 shows examples of this manipulation. Children were presented with an equal number of trials in which: (i) the magnitude of the target dimension across ratio sets was the same (type E in Figure 1, when brown color corresponds to the target flavor), (ii) the magnitude of the non-target dimension across ratio sets was the same (type D in Figure 1, when brown color corresponds to the target flavor), (iii) the total magnitude of the ratio sets was the same (type A in Figure 1), and (iv) the magnitude of the target and non-target dimension, as well as the total magnitude of the ratio sets, were different across ratio sets (types B and C in Figure 1).

This arrangement rendered two types of trials according to whether the side of the target ratio (correct response) aligned with that corresponding to each unidimensional heuristic or not. For instance, when children were tasked to select the chocolaty milkshake, the correct response was congruent with the side showing more chocolate (i.e., more of the target dimension) in trial types A, B, and D; it was congruent with the side showing less milk (i.e., less of the non-target dimension) in trial types A, C, and E; and it was congruent with the side of the larger total (milk+chocolate) in trial types B and D. For simplicity, we will label these congruent (aligned). Conversely, the magnitude of the target dimension was not informative or did not align with the side of the target ratio in trial types E and C, respectively (see Figure 1 above); it was not informative or did not align with the side of the smaller non-target dimension in trial types D and B, respectively; and it was not informative (trial type A) or did not align with the absolute magnitude of the ratio set in trial types C and E. For simplicity, we can label these trials as incongruent trials (not aligned).^3^

In the current study, we followed the analytical approach described in Szkudlarek and Brannon (2021) to investigate whether children’s approach to ratio reasoning is based on specific unidimensional heuristics. Thus, we examined the association between the pattern of errors and the Ratio Deviation from Heuristic Model for each heuristic (whether a specific unidimensional heuristic aligns with the target ratio or not; i.e., congruent and incongruent trials, above). For instance, if errors and incongruent trials for a given heuristic (those that deviate from that heuristic) are both dummy-coded as 1, then, a positive coefficient would indicate that children make incorrect responses on trials where a given heuristic predicts an incorrect response. Put differently, this approach investigates whether the pattern of errors responds to the child’s tendency to focus on specific aspects during ratio reasoning tasks. Table 1 shows the values of the Ratio Deviation from Heuristic Model (RDHM) for each heuristic and type of trial, as depicted in Figure 1, if children were tasked to select the chocolate milkshake. Whereas having different types of trials (in which the target ratio is associated with either the target dimension, non-target dimension, or the absolute magnitude of the ratio set) allows estimating more precisely the child’s ratio reasoning abilities, this analytical approach takes into account that different strategies may trigger a correct response (alignment heuristic-ratio).

**TABLE 1:** RDHM for each heuristic and type of trial in Figure 1.

	RDHM_target	RDHM_non-target	RDHM_absolute magnitude
A	0	0	1
B	0	1	0
C	1	0	1
D	0	1	0
E	1	0	1

Another aim of the current study was to unravel the association between children’s ratio reasoning and math abilities. This is because it is not yet clear that ratio reasoning reflects math abilities in children. For instance, both Gouet et al. (2020) and Szkudlarek and Brannon (2021) found no association between non-symbolic ratio reasoning (using discrete representations) and children’s numeracy skills (as measured with the Key-Math-3) after accounting for individual differences in ANS acuity. In the current study, we expand this research and investigate whether an association emerges between the child’s ratio reasoning skills (and use of unidimensional heuristics) and two math abilities that reflect different numerical knowledge: math problem solving and number line estimation skills. Given that there is evidence that preschool children can communicate about proportions using a (non-labeled) number line (Möhring et al., 2016, 2018), and that number line estimation skills have been directly linked to proportional reasoning (for rationale, see Barth and Paladino, 2011), a stronger association between ratio reasoning skills and number line estimation skills is expected.

## Materials and Methods

### Sample

Data from the current study were drawn from a longitudinal study examining the interplay between ratio reasoning, numerical magnitude processing, and fractions understanding. One-hundred and thirty-two children (Mage = 68 months, range = 62–75, *SD* = 3.5; 48% females) participated in the current study. Children were recruited from three government-operated kindergartens in Singapore and were tested during the first half of the second year in kindergarten (K2; the year children turn 6 years old). We chose this age because there is evidence that these children may operate with continuous representations but fail to operate with discrete representations during ratio reasoning (Jeong et al., 2007).

Children were tested in their preschools, either in a separate room or a corner of their classroom. Testing per child took approximately 1 h, split over two sessions with no more than one session per day (children were assessed on other cognitive and numerical aspects that are not included here such as WM capacity, visuospatial WM, symbolic knowledge, and numerical magnitude processing). The order in which the tasks were administered was counterbalanced across children with the following constraint, the ratio reasoning tasks (continuous and discrete) were presented on different sessions. Parents provided informed written consent prior to the child’s participation and verbal assent from the child was required on the day of testing. All children received a small token of appreciation after completing the tasks. All recruitment and testing procedures were approved by the Institutional Review Board at the university of the first author (protocol number IRB 2019-09-046).

### Materials and Methods

All tasks (with the exception of the Math Problem-Solving subtest) were computerized and run with EPrime. The tasks were implemented on 16” touch screen laptops.

#### Ratio Reasoning Skills (Continuous and Discrete Ratio Reasoning)

**FIGURE 2:**
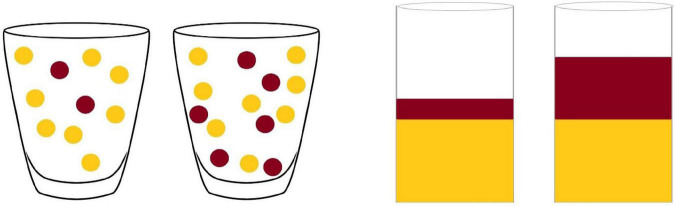
Illustration of one trial in the discrete (left) and continuous ratio reasoning task.

Children were presented with both tasks. In the ratio reasoning tasks, children were presented with a picture of two glasses—each containing a mix of chocolate and milk (or chocolate and banana chips in the discrete task)—and were asked to select the glass corresponding to the chocolate (or milky) flavor in the task with continuous representations, and chocolate (or banana) flavor in the task with discrete representations. Children were randomly assigned to a specific flavor in each task. In the continuous condition, the glasses resembled two stacked bar graphs. In the discrete conditions, the mix in the glasses corresponded to dots of different colors (see Figure 2).

Regardless of the task, first, the child was presented with two slides on how to make milkshakes by mixing two ingredients and how milkshake flavors change as the amount of each ingredient varies. Then, the child was presented with four practice trials corresponding to each of the four types of trials described above (same target dimension, same non-target dimension, same total, and different target/non-target/total). Feedback (right or wrong) was provided during the practice trials, and children were not shown the experimental trials until they successfully responded to three out of four practice trials. Then, children were presented with 48 unique experimental trials. In one-fourth of stimuli, the magnitude of the target dimension was the same across ratio sets, one-fourth showed the same non-target dimension, one-fourth the same total in each ratio set, and one-fourth differed in terms of the magnitude of target/non-target dimension and total magnitude across ratio sets. Half of the trials showed the target ratio to the right.

Given that difficulty with the ratios being processed may affect ratio reasoning, we manipulated the magnitude of each dimension in each ratio set and across ratio sets to minimize the correlation of the ratio comparison corresponding to each unidimensional heuristic (e.g., ratio of the target dimension across ratio sets) with the ratio of the ratio sets. In the current study, the correlation of the ratio of ratios with the ratio corresponding to each unidimensional heuristic was: target dimension, *r* = 0.28, non-target dimension, *r* = 0.25, and ratio total, *r* = 0.10. Thus, the range^4^ of the ratio of ratios in trials showing the same-target dimension, same non-target dimension, same total, and those showing different target/non-target and total magnitude across ratio sets was similar (0.4 and 0.7, 0.3 and 0.7, 0.4 and, 0.6, and 0.3 and 0.9, respectively). The ratio of ratios ranged between 0.3 and 0.9 (e.g., 3:4 | 2:8 and 4:6 | 3:4, respectively). The ratio of target to non-target dimension in each glass varied between 0.01 and 0.08 (e.g., 1:8 and 3:4, respectively). In the discrete condition, the number of dots representing chocolate or banana chips in any ratio set ranged between 1 and 40. All dots had the same diameter and did not vary in other physical properties. The location of dots in each glass was varied randomly. Continuous representations were also scaled. For instance, the area of the ratio set representing 1:2 (ratio 0.5) was half of that of 2:4 (ratio 0.5). We specifically avoided cross-half trials depicting either the target or non-target dimension as more than 0.5 in one ratio set and less than 0.5 in the other one (e.g., 1:3 vs. 5:2) as these trials would be too easy in the continuous version for 6-year-olds. The list of stimuli that were used may be found in Supplementary Material.

In the discrete task, children were instructed not to count the dots. In each trial (both continuous and discrete conditions), a fixation point (3 stars) was shown for 1,000 ms, then, the stimulus was shown for 4,000 ms (or until the child’s input). If no input was received (touch screen), a response screen with two question marks on each side of the screen was shown. Then, children were encouraged to touch the side of the screen corresponding to the assigned target dimension. The order in which trials were presented was pseudorandomized across participants: trials were arranged in four blocks to achieve an equal number of right and left responses as well as a balanced distribution of trials corresponding to the four types described above. The order in which blocks were presented was randomized. In the current sample, Spearman-Brown split-half reliability coefficients were = 0.70 and 0.85 for discrete and continuous ratio reasoning tasks, respectively.

#### Non-symbolic Magnitude Processing Skills (Approximate Number System)

We included a measure of the child’s ANS acuity because the ANS is associated with ratio reasoning skills (Szkudlarek and Brannon, 2021) and may affect the association between math abilities and ratio reasoning abilities. We used a non-symbolic magnitude comparison task. Each trial consisted of the presentation of two arrays of dots which were described to the children as coins to be collected. The arrays were presented for 2 s, after which a screen appeared showing two question marks. The child was asked to indicate which display showed more coins by touching the side of the screen corresponding to the array showing more coins. Feedback was provided after each trial, and after every 25 trials a feedback screen was presented to motivate the child, which indicated they had collected enough treasure for part of the “captain’s boat.” The number of dots of each color in the array varied from 5 to 30, with each pair depicting a ratio difference of 0.91 (e.g., 10:11), 0.83 (e.g., 5:6), 0.77 (e.g., 7:9), 0.71 (e.g., 5:7), or 0.67 (e.g., 6:9). At each ratio level, the absolute difference between the stimulus pairings differed (e.g., at ratio 0.67, stimulus pairings were 6:9, 10:15, 12:18, 18:27, 20:30). Five pairs at each ratio level were presented (50 experimental trials). Half the trials presented the highest number on the left side of the screen. All trials were area controlled, and to ensure children were responding on the basis of quantity and not dot size, individual item size was varied to ensure that items in the less numerous arrays were not always larger than those in the more numerous arrays. Accuracy (% of correct trials) was the measure of non-symbolic magnitude processing. In the current sample, Spearman-Brown split-half reliability coefficient = 0.67.

#### Math Abilities

We used two different measures capturing two aspects that differ in terms of complexity—math problem solving and number line estimation skills.

##### Math Problem-Solving Skills

Children completed the *Math Problem Solving* subtest from the Wechsler Individual Achievement Test—III (WIAT-III; Wechsler, 2009). This subtest is a verbal problem-solving test that (for this age group) measures the ability to count, identify geometric shapes, and solve single and multi-step word problems with the aid of visual cues. Testing was discontinued after the child made 6 consecutive incorrect responses. Raw scores were used as the measure of math achievement. Cronbach’s alpha in the current study was 0.89.

##### Number Line Estimation Skills

Children were tasked to indicate on a line where a number at the top should go. On each problem, a number between 1 and 9 was presented along with a horizontal number line in the middle of a computer screen with 0 at the left end and 10 at the right end. Children completed four practice trials, after which the remaining numbers were presented, one at a time without feedback. All the numbers from 1 to 9 were presented. Each number was presented twice to calculate an average positioning. Number line estimation proficiency was based on the percentage of absolute error (PAE). The PAE was calculated as [(actual number estimated—target number presented)/scale of number line] × 100. A lower PAE indicates better estimation skills. Spearman-Brown split-half reliability coefficient in the current study was = 0.75.

### Analytical Approach

We used a hierarchical linear model (cross-classified model). This is because the data have a hierarchical structure and some relatedness between responses of the same respondent is expected, as well as some relatedness between observations for the same item. The cross-classified approach correctly and reliably partitions the variance in ratio reasoning to differences across items and differences across children. For consistency with previous research, we first examined the role of representation format on the child’s ratio reasoning skills, as well as the association between ratio reasoning and children’s math abilities. To this end, we formulated a probit model in which the probability of ratio reasoning (1 = correct; responses correspond to level 1 in the model) was a linear function of item-level and subject-level variables (see Supplementary Material). At the item level (level 2a), we used task format (1 = discrete) to predict the child’s responses. At this level, we also included the ratio of the ratios to account for difficulties with the ratios being processed. At the subject-level (level 2b), we regressed the child’s responses onto ANS acuity. Furthermore, both math abilities were regressed onto ANS acuity and the child’s ratio reasoning ability to investigate whether ratio reasoning is a meaningful predictor of the child’s math skills after accounting for other skills that can affect ratio reasoning.

In a second set of analyses, we examined children’s use of unidimensional heuristics, whether such use varied as a function of the representation format, and the association with math abilities. We used a similar probit model; however, we considered a level-1 (response-level) variable—the Ratio Deviation from Heuristic Model, as in Szkudlarek and Brannon (2021). For each participant and trial, we indicated whether the side of the target ratio corresponded with that of each unidimensional heuristic (heuristic-ratio congruency). We indicated “1” for incongruent trials in which the side of the target ratio did not correspond with a specific heuristic—deviation from heuristic.

A separate model was conducted for each unidimensional heuristic. In these analyses, we reversed the scores of the ratio reasoning task (1 = incorrect). At the response level (level 1), the child’s responses were regressed onto the variable RDHM (1 = incongruent). This effect was allowed to vary at the item- and subject-level (i.e., random slopes). Subsequently, we included the ratio of the ratios and task format as predictors of both the child’s responses and the association between RDHM and the child’s responses at the item level (level 2a). At the subject level, we investigated whether there was an association between the child’s approach to ratio reasoning and the child’s math ability and ANS acuity (as specified in the model described above). Note that this approach is similar to that described in Szkudlarek and Brannon (2021) and that, in each model, we are testing whether children’s errors respond to a specific unidimensional approach. However, in contrast to Szkudlarek and Brannon (2021), we model item- and subject-level data simultaneously rather than relying on two different sets of analyses in which data are aggregated and the possibility of type I and type II error increases.

All inferential statistical analyses were estimated using the option cross-classified in Mplus (Version 8.6; Muthén and Muthén, 1998–2017) and Bayes estimation with diffuse priors (non-informative). Estimates were adjusted based on all available data and corresponded to the median of the posterior distribution. Bayes does not rely on large-sample theory and provides the whole distribution not assuming that it is normal. Each model was run twice to check convergence. The number of iterations in the second run was increased by at least a factor of two (and a minimum of 5,000 iterations). The default convergence criterion was that the Proportional Scale Reduction (PSR) factor was close to 1 for each parameter. We also assessed model convergence by visual inspection of the Bayes posterior distribution, autocorrelation, and trace plots. We report the 95% Bayesian CI to determine the “significance” of the estimates (a 95% CI that does not include zero is an indication that the estimate is significant).

## Results

Descriptive statistics and zero-order correlations are shown in Table 2.

**TABLE 2:** Descriptive statistics and zero-order correlations.

	RR continuous (Accuracy)	RR discrete (Accuracy)	ANS (Accuracy)	Math problem solving (Raw scores)	Number line (PAE)
Mean	0.69	0.68	0.64	26.52	11.92
SD	0.14	0.12	0.13	4.35	6.31
Skewness	−0.32	−0.37	0.19	−0.27	1.78
Min	0.23	0.35	0.40	16	3
Max	0.98	0.98	0.96	35	47
RR continuous	—				
RR discrete	0.345***	—			
ANS	0.268**	0.275**	—		
Math problem solving	0.283**	0.22*	0.223*	—	
Number line	−0.161	−0.163	−0.37***	−0.253**	—

*p < 0.05, **p < 0.01, ***p < 0.001; RR denotes ratio reasoning.

**FIGURE 3:**
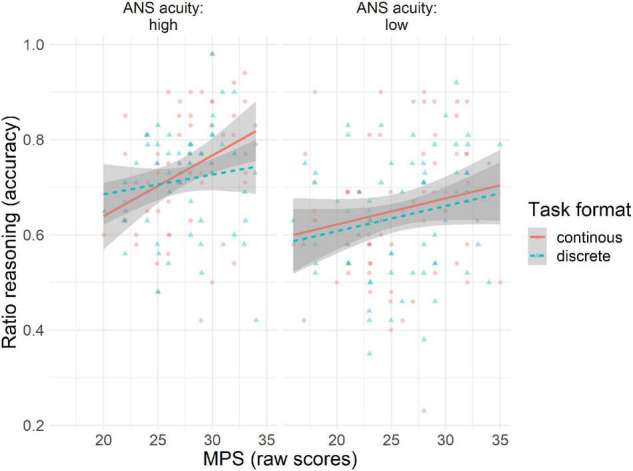
Scatterplots depicting three-way association between ratio reasoning skills (*y*-axis) by task format (colored lines), math problem-solving skills (*x*-axis), and ANS acuity (paneled: high and low correspond to above and below the sample mean, respectively).

The pattern of associations between ratio reasoning skills, math problem solving skills, ANS acuity, and number line estimation skills did not differ across task formats. Figure 3 depicts the three-way association between ratio reasoning, math problem solving, and ANS acuity. It can be observed that children’s math problem-solving skills are more strongly associated with ratio reasoning with continuous representations in children with higher ANS acuity.

### Ratio Reasoning and Task Format

Children performed above chance in both continuous [69%; *t*(131) = 15.63 *p* < 0.001, Cohen’s *d* = 1.36] and discrete [67%; *t*(131) = 16.58 *p* < 0.001, Cohen’s d = 1.45] ratio reasoning tasks. The hierarchical model that was estimated converged well. The overall mean probability of correct ratio reasoning across tasks was 0.70 (at the mean-centered ratio of ratios and ANS acuity). The model did not reveal a main effect of task format (β = −0.06, [−0.210, 0.081]). The ratio of ratios explained variance in the probability of ratio reasoning; the likelihood that more challenging items (those with a ratio of ratios closer to one) were correctly scored was smaller (β = −1.81, [−2.28, −1.32]).

### Ratio Reasoning, Approximate Number System, and Math Abilities

The model revealed an association between ANS acuity and the child’s ratio reasoning skills (β = 0.91, [0.452, 1.38]). Children with higher ANS acuity had better ratio reasoning skills. ANS acuity was also associated with the child’s number line estimation skills (β = −16.84, [−25.67, −8.55]). The negative coefficient indicates that children with better number line estimation skills (lower PAE) had better ANS acuity. Ratio reasoning skills were only associated with math problem solving after accounting for individual differences in ANS acuity (β = 3.90, [1.27, 6.58]). Children with better ratio reasoning skills also had better math problem-solving skills.

### Children’s Approach to Ratio Reasoning and Representation Format

The models that were specified for the various unidimensional heuristics converged well. The parameter estimates of each model are shown in Table 3. In line with the results from the model that was presented above, none of the models revealed a format effect. A ratio effect emerged across models; the probability of error increased as the ratio of ratios approached 1 (more challenging). Note that these were probit models in which the dependent variable reflected probability of error, so the coefficients regarding the effects of difficulty with the ratios being processed and task format show the opposite sign (with respect to those shown in the previous analysis).

**TABLE 3:** Unstandardized parameter estimates (and 95% Bayesian CI) of the unidimensional models.

	Target dimension	Non-target dimension	Absolute magnitude
Threshold	**0.435 (0.311, 0.630)**	**0.761 (0.616, 0.932)**	**0.505 (0.396, 0.603)**
RDHM	**0.771 (0.432, 1.05)**	−**0.770 (**−**1.08,** −0**.481)**	**0.633 (0.424, 0.844)**
RatioR effect	**1.65 (0.882, 2.41)**	**2.54 (1.73, 3.31)**	**1.83 (1.20, 2.47)**
Format effect	0.091 (−0.126, 0.319)	0.043 (−0.193, 0.284)	0.017 (−0.186, 0.221)
^a^RatioR → RDHM	0.208 (−1.09, 1.53)	0.636 (−0.556, 1.79)	0.042 (−0.594, 0.660)
^a^Format → RDHM	−0.132 (−0.497, 0.253)	0.089 (−0.248, 0.440)	−0**.306 (**−**0.507,** −0**.107)**
^a^ANS → RDHM	−0.07 (−1.52, 1.37)	1.21 (−0.215, 0.261)	−0.584 (−2.12, 0.971)
^a^RDHM → Nline	0.558 (−0.695, 1.79)	−0.978 (−2.21, 0.252)	0.970 (−0.059, 1.99)
^a^RDHM → MPS	−0.02 (−0.904, 0.824)	**1.24 (0.387, 2.09)**	−**0.861 (**−**1.57,** −0**.096)**

*Top and bottom panels refer to item-level and subject-level effects, respectively. Parameters in bold indicate that the 95% CI does not cross zero. Ratio R refers to the ratio of ratios. MPS, Math problem solving; NLine, Number line estimation skills. The threshold in the probit regression is estimated at the mean-centered ratio of ratios and subject-level variables. Contrast coding is used to code RDHM (i.e., −0.5 and + 0.5 for congruent and incongruent trials, respectively) and format (i.e., −0.5 and + 0.5 for continuous and discrete representations, respectively) so the threshold corresponds to the overall mean probit.*

*^a^Indicates a cross-level interaction.*

The models revealed that the association between RDHM and the pattern of errors was substantially different from zero. That is, children’s errors responded to specific unidimensional approaches. Nonetheless, this effect was qualitatively different across models. The positive coefficients indicate that children’s patterns of errors reflected an approach based on the magnitude of the target dimension across ratio sets and the absolute magnitude of a ratio set. This is also confirmed by the negative coefficient regarding the model *non-target dimension*—children’s approach was the opposite to focusing on the non-target dimension across ratio sets. The association between RDHM and the pattern of errors was moderated by the format of presentation in the model *absolute magnitude.* The negative coefficient regarding this cross-level interaction indicates that such an approach to ratio reasoning was less evident when it comes to discrete representations (coded as 1).

### Children’s Approach to Ratio Reasoning, Approximate Number System Acuity, and Children’s Math Abilities

The associations between children’s ratio reasoning skills and both math abilities (after accounting for ANS disparities) were similar to those reported above (for clarity, these coefficients are not included in Table 3). The models revealed no association between the approach to ratio reasoning and both ANS acuity and number line estimation skills. That is, difficulty with the ratios being processed and the child’s ability to represent ratios do not affect whether children focus on one or another dimension during ratio reasoning. Similarly, no association between children’s number line estimation skills and the approach to ratio reasoning was found. Nonetheless, the models revealed that the child’s math problem-solving skills are associated with the approach to ratio reasoning. Children with better math skills approach ratio reasoning focusing on the magnitude of the non-target dimension to a larger extent than those with poorer math skills. Furthermore, the negative coefficient for the model that tested whether the pattern of errors reflected children’s focus on the absolute magnitude of the ratio set indicates that this approach is less likely in children with better math skills.

## Discussion

### Kindergarteners’ Approach to Ratio Reasoning

In the current study, we investigated kindergarteners’ ability to reason about ratios that involve discrete entities and continuous amounts. Specifically, we looked at whether the representation format, which can affect the likelihood of encoding the magnitude of each dimension in a ratio set, affected how children approach ratio reasoning. We found that kindergarteners already performed above chance when they were tasked to make ratio judgments between two ratio sets. Interestingly, we did not observe performance differences between continuous and discrete representations. Indeed, accuracy estimates in the discrete task were in line with those reported in other studies that have used a similar task with slightly older children (between 60 and 70%; e.g., Szkudlarek and Brannon, 2021). Note that some studies that have found similar-age children performing below chance when ratio reasoning involves discrete entities (e.g., Jeong et al., 2007; Boyer et al., 2008; Hurst and Cordes, 2018) have used ratio representations that may trigger other cognitive processes such as counting, which may negatively affect ratio reasoning performance (the *whole number bias* or *numerical interference*; Ni and Zhou, 2005).

The fact that children performed similarly with continuous and discrete ratio displays is not congruent with findings that suggest that ratio reasoning with continuous ratio displays would be easier as the cognitive systems involved in processing visual properties that represent ratios develop earlier for continuous magnitudes than for discrete quantities (Kucian et al., 2018). It is also incongruent with the idea that discrete and continuous representations afford different opportunities to establish part–part and part–whole relations. It has been argued that discrete representations pose visual constraints to defining the boundaries of each dimension in a ratio set and to establishing part–part and part–whole relations that are necessary for successful ratio reasoning. In contrast, continuous representations are thought to afford more opportunities to identify the parts/dimensions and the whole and to establish part–whole relations (Mix et al., 2002). Thus, a feasible explanation is that the advantage of continuous representations emerges when children engage in true ratio reasoning and they are able to establish part–whole relations. Indeed, there is evidence that performance with continuous representations improves when the whole is depicted. For instance, Huttenlocher et al. (2002) showed that 2- and 4-year-old children were able to match the size of a target dowel when it was presented in a container but not when it was presented alone. Similarly, Boyer et al. (2008) presented children from kindergarten to Grade 4 with two alternatives of a target proportion (juice-mixture) where the non-proportional alternative showed either the same absolute magnitude as the juice part (i.e., part foil-type) or the same absolute magnitude as the target proportion (i.e., juice + water; whole foil-type); this is equivalent to presenting same-numerator and same-denominator fractions (part foil-type and whole foil-type, respectively). They found that selection of the proportional match when the foil matched the target’s juice part did not exceed chance until third-grade whereas it exceeded chance by kindergarten when the foil matched the target’s total juice + water (i.e., same-denominator fractions were more easily rejected).

In this context, we can assume that children in the current study did not take advantage of the fact that continuous representations provide more visual referents to establish part–whole relations. Put differently, they relied on part–part relations—unidimensional heuristics—that afford similar behavior with continuous and discrete representations. It is worth mentioning that although Boyer et al.’s (2008) findings were interpreted as evidence that children can establish part–whole relations, it is not clear whether children just focused on the magnitude of the target dimension across ratio sets rather than establishing part–whole relations. This is because the non-matching alternatives that were correctly rejected (whole foil-type—same-denominator fractions) do indeed show different denominators and different numerators. For instance, when fractions such as 1/5 and 3/5 (same denominator) are represented visually as stacked bar graphs, we have 1 part of water and 4 parts of juice (ratio 1:4) and 3 parts of water and 2 parts of juice (ratio 3:2), respectively.

Notably, performing above chance does not mean that children engage in true ratio reasoning and establish part–whole relations. The possibility that children engage in unidimensional heuristics during ratio reasoning cannot be completely ruled out because correct responses in ratio reasoning tasks may be because the child either engages in true ratio reasoning or adopts a unidimensional approach. In other words, the target ratio cannot be completely disentangled from any potential unidimensional heuristic (i.e., there are no instances in which the target ratio does not correspond to the ratio set showing either the larger target dimension, the smaller non-target dimension, the larger absolute magnitude, or a combination of these unidimensional approaches). Thus, even if different types of trials are presented and the congruency between the target ratio and each unidimensional heuristic is minimized, children could rely on unidimensional heuristics. For instance, in an attempt to disentangle the child’s behavior during ratio reasoning tasks—unidimensional heuristics vs. true ratio reasoning—, Szkudlarek and Brannon (2021) presented children with a binary discrimination task similar to the discrete condition in the current study. These authors analyzed the strength of the association between the Ratio Deviation from Heuristic Model (as defined above; for instance, a 1 indicated that a specific heuristic did not align with the correct ratio for a particular trial) and the so-called “Child Deviation from Heuristic Model” (which reflected how each child’s actual choices deviated from a specific unidimensional heuristic; for instance, a 1 indicated that the child’s choice in a trial did not reflect that a specific heuristic was being used to solve that trial). With this setting in mind, a perfect correlation (*r* = 1) would indicate perfect accuracy and correlations closer to zero would suggest that the child relied on unidimensional heuristics. Although the authors argued that children engaged in true ratio reasoning since the Pearson correlation coefficients were substantially different from zero, such coefficients were part of a continuum reflecting different degrees of true ratio reasoning and use of unidimensional heuristics, hence, it cannot be concluded that children’s behavior reflected true ratio reasoning. Indeed, those correlation coefficients (Pearson *r* range = 0.35, −0.44) are likely different from one, which reflects that children engaged in unidimensional heuristics to some extent. In other words, only correlations closer to 1 would reflect that children are able to inhibit primitive approaches such as focusing on specific dimensions across ratio sets during ratio reasoning tasks.

In the current study, we found that children relied to a larger extent on the magnitude of the target dimension and the absolute magnitude of the ratio set. This finding aligns with those of studies that have considered children of a similar age range. For instance, Falk et al. (2012) found that children from 4 to 7 years focused on the magnitude of the target dimension whilst older children used additional heuristics—the magnitude of the non-target dimension—to determine the best outcome. Indeed, our findings also align (indirectly) with that possibility as we found that children with better math problem-solving abilities tended to rely more on the magnitude of the non-target dimension than those with poorer math abilities. Note that, Falk et al. (2012) used a discrete task and did not consider whether children focused on the absolute magnitude of the ratio sets.

We also found some support for our hypothesis that the representation format may affect the child’s approach to ratio reasoning as there were some differences between continuous and discrete representations. The interaction between task format and the strategy of focusing on the absolute magnitude of the ratio set suggests that children focused to a larger extent on the absolute magnitude of the ratio set with continuous representations than with discrete representations. However, we are hesitant to suggest that this finding confirms our prediction. In fact, we hypothesized that continuous representations would allow focusing on the magnitude of both target and non-target dimensions since each dimension is perceptually more salient than in discrete representations. One possibility is that such a dual approach to ratio reasoning only emerges at later stages in development when children enter formal education and are exposed to depictions that represent part–whole relations in the context of arithmetic problem solving. It is also argued that focusing on the magnitude on the non-target dimension may be an intermediate but necessary step before children engage in true ratio reasoning and establish part–whole relations (Siegler, 1981). Regarding the interaction that was found, it is feasible that the absolute magnitude of the ratio set was perceptually less salient in the discrete task since the arrays of dots in our study were randomly spaced and the ratio set displaying the larger absolute magnitude (i.e., more dots) did not necessarily represent the larger overall area.

### Kindergarteners’ Ratio Reasoning and Math Abilities

In the current study, we also found that ratio reasoning abilities in kindergarteners are already associated with their math abilities even after accounting for differences in ANS acuity, which is a key explanatory variable associated with ratio reasoning abilities and the development of math abilities (Szkudlarek and Brannon, 2021; for a meta-analysis, see Schneider et al., 2017). We found that math problem solving was related to the child’s ratio reasoning skills. This association is probably reflecting higher-level processing since the Math Problem Solving subtest does not only include single and multi-step problems but tackles other abilities such as the ability to identify geometric shapes. Indeed, Szkudlarek and Brannon (2021) found no association between ratio reasoning and a measure of basic numerical processing. Similarly, we did not find evidence of an association between ratio reasoning and the child’s number line estimation skills, which reflect knowledge of number magnitude and order. Note that this null effect was not expected as there is evidence that children may engage in proportional reasoning during number line estimation (Barth and Paladino, 2011; but see Kim and Opfer, 2017, for a different rationale) and some studies have found that children can efficiently communicate about proportions using a (non-labeled) number line (e.g., Möhring et al., 2016, 2018; Gouet et al., 2020). Thus, as mentioned above, it is feasible that the skills that were measured with the ratio reasoning tasks in the current study reflect another type of higher-order processing.

## Conclusion and Future Studies

Our findings suggest that kindergarteners already follow an additive strategy to ratio reasoning and tend to focus on the magnitude of the target dimension across ratio sets as well as on the absolute magnitude of the ratio set. We found that this approach does not vary substantially across different types of representations that naturally afford different opportunities to estimate the absolute magnitude of each dimension within each ratio set and across ratio sets. Our study also underscores the association between ratio reasoning skills and the child’s math abilities even before children enter formal school and supports children’s stimulation of ratio reasoning abilities early in development. This is in line with educational recommendations that probabilistic reasoning and data analysis are core mathematical aspects that children must develop starting from Pre-K2 (National Council of Teachers of Mathematics, 2010).

Although our findings suggest that kindergarteners rely on specific unidimensional heuristics to reason about ratios, we acknowledge that we cannot address the question of whether they engage in true ratio reasoning. As mentioned above, only perfect accuracy or finding that children can inhibit unidimensional approaches to ratio reasoning would support the possibility that young children engage in true ratio reasoning. Other methodological approaches such as sequential presentation of ratio sets and evidence from eye-tracking may provide clearer insight into that question. More research is also needed to fully understand how non-symbolic ratio reasoning abilities in children contribute to fractions’ understanding later in development. For instance, it has been suggested that a non-symbolic ratio magnitude system might provide support for an understanding of symbolic fractions (Matthews et al., 2016). Nevertheless, that thesis remains barely explored. Similarly, more research is needed to determine how ratio reasoning abilities in young children can be supported and whether specific pedagogies are needed to provide these children with an understanding of proportions and probabilities. Although many studies have investigated this core aspect of the child’s math abilities since the early 60s, few studies have specifically focused on how to support children’s ratio reasoning skills and whether these skills may be incorporated into the suite of diagnostic measures that are frequently used to screen children at risk of mathematics early in development.

## Data Availability Statement

The datasets presented in this study can be found in online repositories. The names of the repository/repositories and accession number(s) can be found below: NIE data repository https://repository.nie.edu.sg/.

## Ethics Statement

The studies involving human participants were reviewed and approved by the NTU Institutional Review Board. Written informed consent to participate in this study was provided by the participants’ legal guardian/next of kin.

## Author Contributions

DM: conceptualization, funding acquisition, supervision, project administration, methodology, formal analysis, data curation, and writing the original draft. RB and PC: conceptualization, funding acquisition, and writing the original draft. JO: writing the original draft. All authors contributed to the article and approved the submitted version.

## Author Disclaimer

Any opinions, findings, conclusions, or recommendations expressed in this material are those of the author(s) and do not necessarily reflect the views of the Singapore MOE and NIE.

## Conflict of Interest

The authors declare that the research was conducted in the absence of any commercial or financial relationships that could be construed as a potential conflict of interest.

## Publisher’s Note

All claims expressed in this article are solely those of the authors and do not necessarily represent those of their affiliated organizations, or those of the publisher, the editors and the reviewers. Any product that may be evaluated in this article, or claim that may be made by its manufacturer, is not guaranteed or endorsed by the publisher.

## Footnotes

1 Note that we use the term ratio reasoning to refer to performance on tasks that require either matching or comparing ratio sets under probabilistic or proportional contexts. Despite the affinity of these terms, and that assessment of probabilities and matching judgments start with comparing ratios, some authors have argued that there are subtle differences (see Falk et al., 2012).

2 “Understanding the multiplicative relationships between rational quantities” (Boyer et al., 2008, p. 1478).

3 Note that children were asked to select one of two flavors, hence, the pattern of incongruent trials for children who were tasked to select the milkier glass would be the opposite; the side of the target ratio would not align with the larger target dimension, smaller non-target dimension, and larger total in trial types B and D, trial types C and E, and trial types A, B, and D, respectively.

4 Note that the ranges corresponding to the same target dimension and same non-target dimension correspond to the non-target dimension and target dimension, respectively, for children assigned to the alternative milkshake flavor.
