# Supplementary material for: Non-symbolic Ratio Reasoning in Kindergarteners: Underlying Unidimensional Heuristics and Relations With Math Abilities
Source: Front Psychol. 2022 Feb 11;13:800977. doi: 10.3389/fpsyg.2022.800977 (PMC8874013; doi:10.3389/fpsyg.2022.800977)
Supplement: Supplementary file 1 [file Data_Sheet_1.docx]

Supplementary Material

# Stimuli and congruency wining ratio and unidimensional heuristic

| **Stimuli (chocolate/milk)** | | **Response (preferred flavor)** | | | | | | | | | | | | |
| --- | --- | --- | --- | --- | --- | --- | --- | --- | --- | --- | --- | --- | --- | --- |
|  | | **Chocolatey flavor** | | | | | | **Milky flavor** | | | | | | |
| **SET A** | **SET B** | | **RC_T** | **RC_NT** | | **RC_TOT** | | | **RC_T** | | **RC_NT** | | **RC_TOT** | |
| 5/15 | 20/30 | | c |  | c | |  | | | c | |  | |  |
| 2/4 | 6/8 | | c |  | c | |  | | | c | |  | |  |
| 1/3 | 3/4 | | c |  | c | |  | | | c | |  | |  |
| 4/6 | 1/3 | | c |  | c | |  | | | c | |  | |  |
| 15/20 | 5/15 | | c |  | c | |  | | | c | |  | |  |
| 30/40 | 10/20 | | c |  | c | |  | | | c | |  | |  |
| 15/20 | 20/30 | |  | c |  | | c | | |  | | c | |  |
| 1/2 | 2/6 | |  | c |  | | c | | |  | | c | |  |
| 3/4 | 2/8 | | c | c |  | | c | | | c | | c | |  |
| 4/6 | 3/4 | |  | c |  | | c | | |  | | c | |  |
| 10/40 | 15/20 | | c | c |  | | c | | | c | | c | |  |
| 10/30 | 5/10 | |  | c |  | | c | | |  | | c | |  |
| 5/20 | 15/20 | | c |  | c | |  | | | c | |  | |  |
| 5/15 | 10/15 | | c |  | c | |  | | | c | |  | |  |
| 5/20 | 10/20 | | c |  | c | |  | | | c | |  | |  |
| 2/8 | 6/8 | | c |  | c | |  | | | c | |  | |  |
| 2/6 | 4/6 | | c |  | c | |  | | | c | |  | |  |
| 2/4 | 3/4 | | c |  | c | |  | | | c | |  | |  |
| 2/4 | 1/4 | | c |  | c | |  | | | c | |  | |  |
| 2/3 | 1/3 | | c |  | c | |  | | | c | |  | |  |
| 3/4 | 1/4 | | c |  | c | |  | | | c | |  | |  |
| 15/20 | 10/20 | | c |  | c | |  | | | c | |  | |  |
| 20/30 | 10/30 | | c |  | c | |  | | | c | |  | |  |
| 30/40 | 10/40 | | c |  | c | |  | | | c | |  | |  |
| 10/15 | 10/30 | |  | c |  | | c | | |  | | c | |  |
| 2/3 | 2/8 | |  | c |  | | c | | |  | | c | |  |
| 5/15 | 5/20 | |  | c |  | | c | | |  | | c | |  |
| 5/10 | 5/15 | |  | c |  | | c | | |  | | c | |  |
| 2/4 | 2/8 | |  | c |  | | c | | |  | | c | |  |
| 2/6 | 2/8 | |  | c |  | | c | | |  | | c | |  |
| 1/3 | 1/2 | |  | c |  | | c | | |  | | c | |  |
| 1/4 | 1/3 | |  | c |  | | c | | |  | | c | |  |
| 2/6 | 2/3 | |  | c |  | | c | | |  | | c | |  |
| 10/40 | 10/30 | |  | c |  | | c | | |  | | c | |  |
| 10/40 | 10/20 | |  | c |  | | c | | |  | | c | |  |
| 10/40 | 10/15 | |  | c |  | | c | | |  | | c | |  |
| 10/35 | 20/25 | | c | c |  | | c | | | c | |  | |  |
| 10/40 | 20/30 | | c | c |  | | c | | | c | |  | |  |
| 15/35 | 20/30 | | c | c |  | | c | | | c | |  | |  |
| 2/5 | 3/4 | | c | c |  | | c | | | c | |  | |  |
| 1/4 | 2/3 | | c | c |  | | c | | | c | |  | |  |
| 1/8 | 2/7 | | c | c |  | | c | | | c | |  | |  |
| 4/6 | 3/7 | | c | c |  | | c | | | c | |  | |  |
| 4/6 | 2/8 | | c | c |  | | c | | | c | |  | |  |
| 4/5 | 2/7 | | c | c |  | | c | | | c | |  | |  |
| 10/35 | 5/40 | | c | c |  | | c | | | c | |  | |  |
| 10/15 | 5/20 | | c | c |  | | c | | | c | |  | |  |
| 15/20 | 10/25 | | c | c |  | | c | | | c | |  | |  |

*Note:* c indicates congruency winning ratio and unidimensional heuristic. RC_T: congruency target ratio and target dimension (more-good strategy); RC_NT: congruency target ratio and non-target dimension (less-good strategy); RC_TOT: congruency target ratio and absolute magnitude of the ratio set (more items strategy).

# Models that were estimated

First set of analyses

$$P\left( u_{ⅈjk}=\left. 1 \right|B_{ijk} \right)=\pi_{ijk} (1)$$

$$\Phi^{-1}\left[ \pi_{ijk} \right]=probit\left( \pi_{ijk} \right) (2)$$

$$probit\left( \pi_{ijk} \right) ={\beta_{0}}+\varepsilon_{ijk}, \left( 3 \right)$$

$$\beta_{0}= {{\gamma00}+ \gamma01}w_{j}+{{\gamma02}z_{j}+{\gamma03}t_{k}+\xi}_{j}+ \xi_{k,} \left( 4 \right)$$

$$M_{k}= {\theta00}{+ {\theta01}t}_{k}+{\theta02}\beta_{0}+\delta_{k,} \left( 5 \right)$$

$$N_{k}= {\theta10}{+ {\theta11}t}_{k}+{{\theta12}\beta_{0}}+\upsilon_{k,} \left( 6 \right)$$

Second set of analyses

$$P\left( u_{ⅈjk}=\left. 1 \right|B_{ijk} \right)=\pi_{ijk} (1)$$

$$\Phi^{-1}\left[ \pi_{ijk} \right]=probit\left( \pi_{ijk} \right) (2)$$

$$probit\left( \pi_{ijk} \right) ={\beta_{0}}+\beta_{1} x_{ijk}+\varepsilon_{ijk}, \left( 3 \right)$$

$$\beta_{0}= {{\gamma00}+ \gamma01}w_{j}+{{\gamma02}z_{j}+{\gamma03}t_{k}+\xi}_{j}+ \xi_{k,} (4)$$

$$\beta_{1}= \gamma10+{\gamma11}w_{j}+{{\gamma12}z_{j}+{\gamma13}t_{k}+\tau}_{j}+ \tau_{k,} (5)$$

$$M_{k}= {\theta00}{+ {\theta01}t}_{k}+{\theta02}\beta_{0}+{\theta03}\beta_{1} +\delta_{k,} \left( 6 \right)$$

$$N_{k}= {\theta10}{+ {\theta11}t}_{k}+{{\theta12}\beta_{0}}+{\theta13}\beta_{1} +\upsilon_{k,} \left( 7 \right)$$

*Note:* x, w, z, and t represent variables RDHM, format, ratio of ratios, and ANS acuity, respectively. M and N represent variables math problem solving and number line estimation skills, respectively. j and k indices indicate item- and subject-level variables, respectively.
